# Depressive Symptoms in Women with Gestational Diabetes Mellitus: The LINDA-Brazil Study

**DOI:** 10.1155/2017/7341893

**Published:** 2017-06-08

**Authors:** Patrícia Damé, Kadhija Cherubini, Pâmella Goveia, Geórgia Pena, Leony Galliano, Cristina Façanha, Maria Angélica Nunes

**Affiliations:** ^1^Postgraduate Program in Epidemiology, School of Medicine, Federal University of Rio Grande do Sul, Porto Alegre, RS, Brazil; ^2^Postgraduate Program in Physical Education, Superior School of Physical Education, Federal University of Pelotas, Pelotas, RS, Brazil; ^3^Integrated Center for Diabetes and Hypertension, Ceará State Health Department, Fortaleza, CE, Brazil; ^4^School of Medicine, Unichristus University Center, Fortaleza, CE, Brazil

## Abstract

This study aimed to assess the frequency and severity of depressive symptoms and their relationship with sociodemographic characteristics in women with gestational diabetes mellitus (GDM) who participated in the LINDA-Brazil study. We conducted cross-sectional analyses of 820 women with GDM who were receiving prenatal care in the public health system. We conducted structured interviews to obtain clinical and sociodemographic information and applied the Edinburgh Postnatal Depression Scale (EPDS) to assess depressive symptoms. We classified the presence and severity of depressive symptoms using scores of ≥12 and ≥18, respectively. We used Poisson regression to estimate prevalence ratios (PR). Most of the women lived with a partner (88%), 50% were between 30 and 39 years old, 39% had finished high school, 39% had a family income of 1-2 minimum wages, and 47% were obese before their pregnancies. The presence of depressive symptoms was observed in 31% of the women, and severe depressive symptoms were observed in 10%; 8.3% reported self-harm intent. Lower parity and higher educational levels were associated with lower EPDS score. Depressive symptoms were common and frequently severe among women with GDM, indicating the need to consider this situation when treating such women, especially those who are more socially vulnerable. This trial is registered with NCT02327286, registered on 23 December 2014.

## 1. Introduction

Pregnancy is one of life's most important events, involving social, psychological, and hormonal changes [1]. It is also a time of great vulnerability to the development of mental disorders, especially depressive disorders [2]. Maternal depression has been linked to negative health-related behaviors and associated with morbidity and mortality [2]. This disorder is a major health problem for women of all nationalities. In a review of depression in pregnant women, prevalence rates were found to be 7.4%, 12.8%, and 12.0% in the first, second, and third trimesters, respectively [1]. It is estimated that 25% to 35% of women have depressive symptoms during pregnancy, and up to 20% of them may meet the criteria for major depression in low- and middle-income countries [3]. In a systematic review of 51 studies (with 48,904 participants), 20 of which were conducted in low- and middle-income countries, depression ranged from 17.3% to 57% [2]. Studies in Brazil showed prevalence ranging from 17.3% to 37.9% [2].

Studies that have aimed to assess depression in women with gestational diabetes are rare in the literature. Gestational diabetes mellitus (GDM), a hyperglycemic state detected during pregnancy, is an established risk factor for diabetes [4]. Diabetes is a major cause of morbidity and mortality and is one of the four main chronic diseases identified by the World Health Organization as a focus for prevention and control [5]. GDM also causes adverse outcomes and is related to depressive symptoms, which may affect adherence to treatment and compound pregnancy outcomes. Since GDM and depressive symptoms may have adverse effects on mothers and their children, it is important to investigate the factors related to the presence of depressive symptoms among women with gestational diabetes.

Thus, this study aimed to assess the frequency and severity of depressive symptoms according to the Edinburgh Postnatal Depression Scale (EPDS) and their relationship to clinical and sociodemographic characteristics in the women with GDM who participated in the LINDA-Brazil study.

## 2. Methods

### 2.1. Setting and Sample

We studied 820 women with GDM who were receiving care at tertiary prenatal care facilities within the Brazilian National Health System in 2014 and 2015 in three cities: Porto Alegre, Fortaleza, and Pelotas. This was a cross-sectional study developed during the recruitment for a large clinical trial called LINDA-Brazil, a multicenter-randomized clinical trial that aimed to evaluate the effectiveness of a lifestyle intervention program in preventing type 2 diabetes after pregnancies complicated by GDM in women identified as being at higher risk. The design of the LINDA-Brazil study has been described elsewhere [4].

Eligibility criteria included being 18 years or older, having been diagnosed with GDM, and living within an easily accessible distance to the trial sites, which were located in urban and rural areas of Fortaleza, Pelotas, and Porto Alegre and their metropolitan areas. During the first contact, the study's protocol was explained and an initial signed consent form was obtained.

### 2.2. Measurements

Data on sociodemographic and anthropometric characteristics as well as gestational age, obstetric history (abortion and parity), and GDM diagnosis and treatment were obtained through interviews at the prenatal clinics and reviews of medical records. Socioeconomic status was determined using two indicators: educational level and household income. We used multiples of the minimum wage to measure household income. The minimum wage is the lowest amount an employer can legally pay employees per month. In Brazil, one minimum wage is equivalent to 300 U.S. dollars.

Depressive symptoms during the previous seven days were assessed using the Edinburgh Postnatal Depression Scale (EPDS) and were rated on a scale of 0–30. The EPDS is one of the most widely used screening questionnaires for pregnant and postpartum women and has been validated to Brazilian Portuguese [6].

We conducted interviews during gestation at referral centers for women with high-risk pregnancies before or after their doctor appointments. Structured questionnaires were applied face-to-face by trained interviewers. The questionnaires took approximately 20 minutes to complete, and it took about five minutes to apply the EPDS, which consists of a ten-question questionnaire and is supported by a card with multiple-choice answers to avoid constraints.

A diagnosis of gestational diabetes was made according to the diagnostic criteria used at each center. The criteria were frequently based on a two-step approach (a fasting plasma glucose test followed by an oral glucose test). Initially, diagnoses were often made using a single elevated two-hour plasma glucose test. However, more recently, the criteria developed by the International Association of Diabetes and Pregnancy Study Group have become more commonly used.

### 2.3. Outcome

A score ≥ 12 was used to classify the presence of clinically relevant symptoms. A score ≥ 18 indicated severity of the symptoms. The EPDS included a specific question to assess self-harm intent.

### 2.4. Data Management and Analysis

Poisson regression with robust variance [7] was performed to estimate prevalence ratios (PR). Adjusted prevalence ratios included remaining variables. We defined the presence and severity of symptoms as dependent variables. SAS 9.4 was used for all statistical analyses, using 95% confidence intervals (95% CI).

### 2.5. Ethical Aspects

All participants were informed of the research protocol and signed informed consent forms. The study was approved by the Ethics in Research Committee of the Hospital de Clínicas de Porto Alegre (Project 120097, May 4, 2012).

## 3. Results

Half of the women (50%) were 30 to 39 years old and most of them lived with a partner (88%) and were of low socioeconomic status. The average gestational age was 31 weeks. Only 39% had finished high school and 39% had a family income of 1-2 minimum wages. Many women were obese before their pregnancies (47%). At the time of the interview, 12% were using insulin to treat their GDM, 53% had had one or two prior C-sections and normal delivery, and 71% had no history of abortion. Thirty-one percent of women evaluated showed depressive symptoms, and approximately 10% showed severe symptoms. Furthermore, 8.3% had thought about harming themselves during the previous seven days.  Table 1 shows the frequencies found of the presence and severity of depressive symptoms and self-harm intent.

Adjusted prevalence ratios showed that higher educational and lower parity levels were inversely associated with frequency of depressive symptoms. After adjustment, household income, age, living with a partner, prepregnancy body mass index, abortion, and insulin use during pregnancy were not associated with depressive symptoms in any level (Table 2).

## 4. Discussion

Our study highlights the prevalence of depressive symptoms (31%), their severity (10%), and the frequency of self-harm intent (8.3%) in women with GDM who received tertiary prenatal care. Most of the women were of low socioeconomic status, and many of them were obese before their pregnancies, which show that this population is at greater risk for type 2 diabetes and other chronic diseases. Higher educational and lower parity levels were associated with lower frequency of depressive symptoms in this population.

Studies of depression in women with GDM are scarce in the literature, and most of them have evaluated prepregnancy diabetes or postpartum depression [8, 9] in contrast to the present study, which deals with data from the gestational period. Also, it bears highlighting that different instruments and cut-off scores are used in literature to evaluate depressive symptoms. This may explain the differences found in prevalence and may hinder comparisons of the findings.

Still, the gestational trimester during which the data collection occurred should be observed. A longitudinal study using the Center for Epidemiologic Studies-Depression (CES-D) score pointed out that there are differences in the prevalence of depressive symptoms according to gestational trimester [10]. The data show a slight decrease in the prevalence of depressive symptoms from the first (15%) to the second trimester (14%) but a relevant increase from the second to the third trimester (30%) [10]. In the LINDA-Brazil study, the mean gestational age in the data collection corresponded to the third trimester (31 weeks), which may explain the higher prevalence of depressive symptoms that was found. In contrast, an evaluation of 137 pregnant women with prediabetes that used the Hospital Anxiety and Depression Scale (HADS) indicated that there was no difference in depressive symptoms between the first and last gestational trimesters [8].

It has been estimated that the prevalence of depression in women with GDM ranges from 4.1% to 80.0%, according to a recent systematic review that included 16 studies. The high variability of the results is due to differences among populations and when the data were collected, in addition to the factors that were mentioned above [11]. Furthermore, no specific studies were found on GDM and depression in the Brazilian population.

Moreover, a systematic review [1] identified that studies using an EPDS score of ≥12 (as was used in our study) in the general population of pregnant women showed lower levels of depression (5% to 8.7%) [12, 13] compared to studies conducted with women of low socioeconomic status (29% to 51%), even considering higher EPDS cut-off points (scores of ≥13 and ≥14) [14, 15]. In low- and middle-income countries like Brazil, maternal depression remains underrecognized and undertreated and is related to negative health outcomes, increasing the risk of comorbidities [2] such as GDM [16, 17].

Some studies have evaluated or reviewed depression and sociodemographic aspects, as this study did [2, 18–20]. A review of these aspects showed that age, marital status, and income have an impact on depression [1], in contrast to what was observed in the LINDA-Brazil study, which identified only higher educational and lower parity levels as being inversely associated with depressive symptoms. Importantly, there are also indications that women with histories of depression are more likely to have GDM [20]. However, the present study did not consider this issue.

Low socioeconomic and educational status, inadequate social support, and a history of mental illness have been consistently identified as risk factors for antepartum and postpartum depression in low- and middle-income countries [2]. A recent systematic review of 104 studies on maternal depression showed frequencies ranging from 16.2% to 24.3% in Brazilian studies and those that used the EPDS to evaluate depressive symptoms. These findings support the hypothesis that differences in depression rates may reflect demographic differences and the different instruments used to estimate or classify depression [21].

Regarding self-harm intent, many studies have related it to suicidal ideation. In this context, frequencies ranged from 2.6% to 27.8% [2]. However, this study used one specific question in the EPDS questionnaire to assess self-harm intent in the previous week, illustrating that the ability to compare these data with those of other studies is limited.

One of the limitations of this study is that we collected data only in the third trimester (at a mean of 31 weeks) and not in other gestational trimesters or before delivery. Neither did we investigate the women's social support systems or whether they had histories of depression. It was not possible to make comparisons with pregnant women without diabetes since all of the women studied had GDM. Finally, the EPDS only evaluates depressive symptoms and does not diagnose depression. Thus, it tends to overestimate depression prevalence [18]. Furthermore, differences in cultural and economic factors may be related to under- or overreporting depressive symptoms. Another issue that merits discussion is the different diagnostic criteria used for the study population. Some of the patients were diagnosed using fasting glycaemia while others underwent a complex procedure that included one or two glucose tolerance tests according to a two-step diagnostic procedure. This study was not able to evaluate whether this difference affected the prevalence of depressive symptoms found in the study population.

Screening for depression (especially during early pregnancy) may be important at different times. Further research on women with GDM would be useful to determine whether depression is a substantial risk factor for GDM and to explore other negative outcomes. Such studies could potentially decrease the costs of maternal and neonatal care and reduce short- and long-term morbidity.

## 5. Conclusion

This study found a high proportion of pregnant women with GDM who presented depressive symptoms. Such a frequency is worrisome, given its known negative impact on pregnancy outcomes. Our results suggest the need to consider depressive symptoms in the treatment of women whose pregnancies are complicated by gestational diabetes, especially in more vulnerable populations.

## Figures and Tables

**Table 1:** Frequency of depressive symptom scores obtained using the Edinburgh Postnatal Depression Scale, according to sociodemographic characteristics (*n* = 820).

Variables	Score			Self-harm intent *n* (%)
	<12 *n* (%)	≥12 *n* (%)	≥18 *n* (%)	
*Total*	563 (69)	257 (31)	81 (9.9)	68 (8.3)
*Age (years)*				
18 to 29	223 (70)	97 (30)	31 (10)	26 (8)
30 to 39	279 (68)	132 (32)	39 (9)	34 (8)
≥40	60 (69)	27 (31)	11 (13)	8 (9.2)
*Living with a partner*				
Yes	511 (71)	214 (29)	67 (9)	57 (8)
No	52 (55)	43 (45)	14 (15)	11 (12)
*Education*				
Complete/incomplete university degree	73 (84)	14 (16)	6 (7)	3 (4)
Complete high school	230 (73)	86 (27)	22 (7)	18 (6)
Incomplete high school	140 (63)	81 (37)	30 (14)	29 (13)
Incomplete elementary school	120 (61)	76 (39)	23 (12)	18 (9)
*Household income (minimum wages)*				
≥3	92 (77)	27 (23)	4 (3)	2 (2)
2 to <3	133 (76)	42 (24)	10 (6)	7 (4)
1 to <2	210 (66)	107 (34)	43 (14)	28 (9)
<1	120 (61)	77 (39)	23 (12)	28 (14)
*Prepregnancy body mass index*				
<25 kg/m^2^	126 (70)	55 (30)	17 (9)	14 (8)
Overweight	167 (69)	74 (31)	22 (9)	20 (8)
Obese class I	145 (66)	74 (34)	23 (11)	17 (8)
Obese class II/III	106 (69)	47 (31)	16 (11)	15 (10)
*Insulin use during pregnancy*				
No	500 (69)	224 (31)	74 (10)	64 (9)
Yes	63 (66)	33 (34)	7 (7)	4 (4)
*Parity*				
0	140 (76)	44 (24)	10 (5)	11 (6)
1 to 2	260 (69)	117 (31)	40 (11)	30 (8)
≥3	87 (58)	64 (42)	18 (12)	20 (13)
*Abortions*				
0	355 (70)	150 (30)	43 (9)	42 (8)
1 to 3	128 (64)	73 (36)	25 (12)	19 (9)
≥4	4 (57)	3 (43)	1 (14)	0 (0)

**Table 2:** Association between sociodemographic characteristics and the Edinburgh Postnatal Depression Scale (*n* = 820).

Variables	EPDS ≥ 12		EPDS ≥ 18	
	PR (95% CI)^∗^	*p*	PR (95% CI)^∗^	*p*
*Age (years)*				
18 to 29	1.0	—	1.0	—
30 to 39	1.0 (0.8–1.3)	0.913	0.8 (0.5–1.3)	0.375
≥40	0.9 (0.6–1.3)	0.497	1.0 (0.5–2.2)	0.931
*Living with a partner*				
Yes	1.0	—	1.0	—
No	1.3 (0.9–1.7)	0.099	1.5 (0.8–3.0)	0.232
*Education*				
Complete/incomplete university degree	1.0	—	1.0	—
Complete high school	1.5 (0.8–2.7)	0.156	0.7 (0.3–1.7)	0.404
Incomplete high school	1.9 (1.0–3.4)	<0.05	1.3 (0.5–3.3)	0.647
Incomplete elementary school	1.9 (1.0–3.5)	<0.05	0.9 (0.3–2.5)	0.768
*Household income (minimum wages)*				
≥3	1.0	—	1.0	—
2 to <3	1.3 (0.8–2.1)	0.343	2.9 (0.7–12.8)	0.151
1 to <2	1.6 (1.0–2.5)	0.058	5.7 (1.4–23.3)	<0.05
<1	1.5 (0.9–2.5)	0.082	3.7 (0.8–16.4)	0.086
*Prepregnancy body mass index*				
<25 kg/m^2^	1.0	—	1.0	
Overweight	1.1 (0.8–1.4)	0.757	1.0 (0.5–1.9)	0.971
Obese class I	1.1 (0.8–1.5)	0.701	1.1 (0.6–2.2)	0.720
Obese class II/III	0.9 (0.7–1.3)	0.713	0.9 (0.4–1.9)	0.820
*Parity*				
0	1.0	—	1.0	—
1 to 2	1.3 (0.9–1.8)	0.150	2.1 (1.0–4.5)	0.065
≥3	1.6 (1.1-2.3)	<0.05	2.1 (0.8–5.3)	0.130
*Abortions*				
0	1.0	—	1.0	—
1 to 2	1.2 (0.9–1.5)	0.205	1.4 (0.8–2.3)	0.226
≥3	1.3 (0.8–2.2)	0.324	1.5 (0.5–4.6)	0.516

^∗^Adjusted prevalence ratios estimated using robust Poisson regression, including the table's remaining variables.
